# Method for enhancing single-trial P300 detection by introducing the complexity degree of image information in rapid serial visual presentation tasks

**DOI:** 10.1371/journal.pone.0184713

**Published:** 2017-12-28

**Authors:** Zhimin Lin, Ying Zeng, Li Tong, Hangming Zhang, Chi Zhang, Bin Yan

**Affiliations:** 1 China National Digital Switching System Engineering and Technological Research Center, Zhengzhou, China; 2 Key Laboratory for NeuroInformation of Ministry of Education, School of Life Science and Technology, University of Electronic Science and Technology of China, Chengdu, China; National University of Defense Technology College of Mechatronic Engineering and Automation, CHINA

## Abstract

The application of electroencephalogram (EEG) generated by human viewing images is a new thrust in image retrieval technology. A P300 component in the EEG is induced when the subjects see their point of interest in a target image under the rapid serial visual presentation (RSVP) experimental paradigm. We detected the single-trial P300 component to determine whether a subject was interested in an image. In practice, the latency and amplitude of the P300 component may vary in relation to different experimental parameters, such as target probability and stimulus semantics. Thus, we proposed a novel method, Target Recognition using Image Complexity Priori (TRICP) algorithm, in which the image information is introduced in the calculation of the interest score in the RSVP paradigm. The method combines information from the image and EEG to enhance the accuracy of single-trial P300 detection on the basis of traditional single-trial P300 detection algorithm. We defined an image complexity parameter based on the features of the different layers of a convolution neural network (CNN). We used the TRICP algorithm to compute for the complexity of an image to quantify the effect of different complexity images on the P300 components and training specialty classifier according to the image complexity. We compared TRICP with the HDCA algorithm. Results show that TRICP is significantly higher than the HDCA algorithm (Wilcoxon Sign Rank Test, p<0.05). Thus, the proposed method can be used in other and visual task-related single-trial event-related potential detection.

## Introduction

The increasing demand for computer images and storage has resulted in abundant image data. Computer vision (CV) plays a remarkable role in current image retrieval because of its increasing computer processing speed. Although CV has been successfully applied in image retrieval, these achievements are limited to special conditions. The effective presentation for the interested image is difficult in image retrieval. Human vision (HV) is superior to CV in terms of its robust and general purpose image recognition ability. HV can also easily recognize target images with large variations. Moreover, HV processing time on a recognition task can be as fast as a few milliseconds, because event-related potential (ERP) has rapid specific response after onset of stimulus [1]. A brain–computer interface (BCI) is a state-of-the-art human–machine interaction technology [2, 3]. This interface records signals of human brain activity (e.g., electroencephalogram) to analyze human intention and then sends the results to a computer. The P300 component in an EEG signal can be used as an indicator to categorize the interest of a user [4]. Moreover, the P300 is a common ERP component and shows a peak waveform when small probability events are observed after approximately 300–500 ms [5]. And many scholars are build a variety of BCI system using the P300 and other EEG components. Erwei Yin et al. combine the P300 component and steady-state visually evoked potential (SSVEP) to build a high-performance hybrid BCI speller system[6–8]. Dewen Hu et al. used the P300 component to construct an auditory / tactile visual saccade-independent P300 BCI system[9]. These P300 components can be exploited when building a target-image detector based on rapid serial visual presentation (RSVP) paradigm [10, 11].

The P300 component exhibits significant waveform characteristics in the time domain. Thus, P300 can be extracted through average multiple trials of EEG signals. In particular, some parameters of P300 component, such as latency and amplitude, are not fixed, but these parameters are important in evaluating P300. In [12], the latency and amplitude of the ERP may vary over time for a given task in relation to different experimental parameters, such as target probability and stimulus meaning. Parra et al. proposed the hierarchical discriminant component analysis (HDCA) algorithm [13–16] to overcome the temporal variability of latency and amplitude. This group separated single-trial EEG signals into several time windows and calculated the spatial filter to maximize the separation between target and nontarget categories. Alpert et al. proposed the hierarchical discriminant principal component analysis (HDPCA) algorithm[17], which introduces the principal component analysis for dimensionality reduction. Marathe et al. developed the sliding HDCA (sHDCA) algorithm [18, 19]. These methods are often focused on the EEG aspects.

Several scholars have considered the combination of EEG and CV to enhance the recognition accuracy. Sajda et al. proposed a closing the loop in cortically-coupled CV (close-loop 3CVision) system to detect the category of subject’s interest image [13, 16]. In their system, the score of the subject interest in an image was estimated by the HDCA algorithm [14], and the combination of this score and CV infers the interest image category from a large database. Wang et al. proposed a similar closed-loop system for face retrieval by coupling EEG-based target image labeling and CV-based label propagation [20]. These techniques involve decision-level fusion that first calculates the EEG interest score, and then CV combines the interest score to guess the user’s target of interest. We believe that the CV can be further integrated in the calculation of the interest score to obtain better results.

We propose a novel method for target recognition in which we can acquire a priori estimate of the deformation of the P300 component of the target image by estimating image complexity (IC) and train the classifier to improve the overall performance. Early studies [5, 12, 17–19, 21, 22] have shown that the specific content of an image will affect the P300 component amplitude and latency. In this study, we used the deep neural network (DNN) to extract image semantic and pixel information [23, 24] and quantify the IC. Moreover, we trained the classifiers separately according to the different complexity ranges. During testing, we used all classifiers and synthesized the results to arrive at a final score. We called this process as a priori image recognition algorithm of IC, that is, Target Recognition using Image Complexity Priori (TRICP) algorithm. We compared TRICP with HDCA algorithm under different classifier parameters, and the results show that TRICP is significantly higher than the HDCA algorithm (Wilcoxon Sign Rank Test, p<0.05).

## Methods

### Participants

A total of 19 subjects (16 males and 3 females, age range of 21 to 24, and right-handed) participated in the experiment. All subjects were students of Zhengzhou University and did not have any previous training in the task, and all participants were recruited in January 2016. The subjects exhibited normal or corrected-to-normal vision with no neurological problems and were financially compensated for their participation. This study was conducted after we obtained informed consent and Ethics Committee approval of China National Digital Switching System Engineering and Technological Research Center. All of the participants provided their written informed consent to participate in this study.

### Visual stimuli and procedure

**Fig 1:**
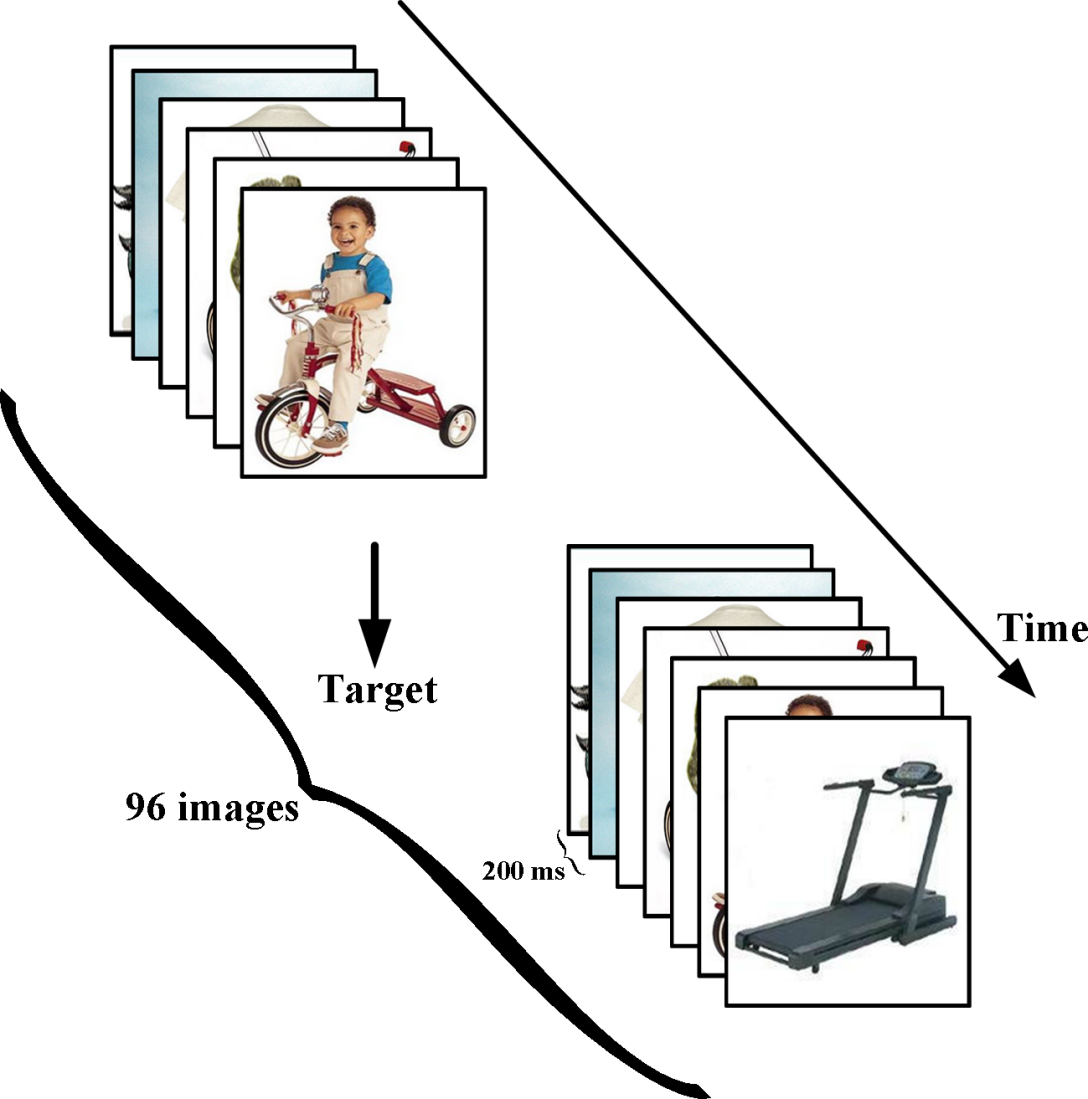
Rapid serial visual presentation (RSVP) paradigm. The RSVP sequence consisted of 25 blocks (a total of 2400 images, i.e., 25 target categories with 300 images and 175 nontarget categories with 2100 images), and each block comprised one target category (12 images) and seven nontarget categories (84 images). The images are presented in 25 blocks, with a distinct target category in each block. The target categories of each block are shown in Table 1. Each image is presented for 200 ms (The image is similar but not identical to the original image, and is therefore for illustrative purposes only).

The participants were seated 75 cm in front of a monitor. Images were chosen from the Caltech-256 database [25] and presented to the subjects using the RSVP paradigm [10, 26]. The images were shown in blocks of 96 and flashed at 5 Hz (Fig 1). Each image was positioned at the center of the computer monitor. A fixation cross was flashed immediately prior to the presentation of each block to allow the users to focus their gaze on the images during the RSVP sequences. For these tasks, the RSVP sequence consisted of 25 blocks (a total of 2400 images, i.e., 300 target images from 25 categories and 2100 non-target images from 175 categories). Each block consisted of 12 target images from one category and 84 non-target images from seven categories (12 images in each category). The target categories for each block differed from one another and are shown in Table 1.

**Table 1:** Target categories for the 25 blocks.

Block	Target	Block	Target	Block	Target
1	Cup	10	Pan	19	Photocopier
2	Butterfly	11	Helicopter	20	Rainbow
3	Camel	12	Sandglass	21	Superman
4	Centipede	13	Lizard	22	Shoes
5	Displayer	14	Kangaroo	23	Football
6	Carriage	15	Cheetah	24	Bicycle
7	Dolphin	16	Owl	25	Unicorn
8	Glasses	17	Minotaur		
9	Helmet	18	Paper-clip		

### System overview

**Fig 2:**
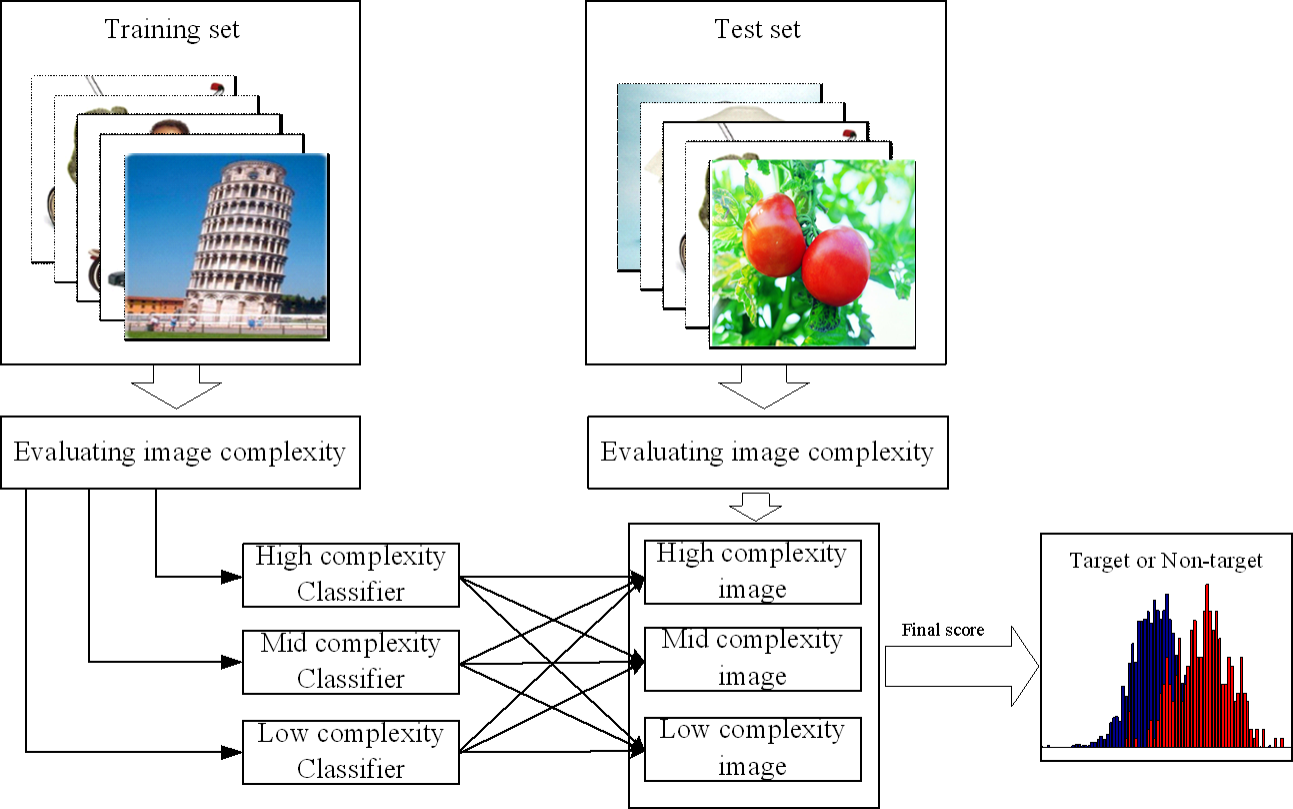
System overview. First, the sample data are divided into equal three parts according to the image complexity (IC). The EEG data induced by high-, medium-, and low-complexity images. We trained the classifiers separately on the different data sets. During testing, we first determined the complexity and category (high-, medium-, or low-complexity image) of the test picture. Then, we calculated the interest scores of the EEG induced by a test image using the corresponding classifier. The result was combined with a certain weight to obtain the final decision score (The image is similar but not identical to the original image, and is therefore for illustrative purposes only).

In this paper, we propose a TRICP method for image retrieval (Fig 2). The algorithm includes three major components, namely, CV, EEG, and mix modules. First, we used the CV module to estimate IC. We sorted the IC score and divided the images into three categories, namely, high-, medium-, and low-complexity images. Then, we recoded all image EEG data and trained three corresponding EEG classifiers (high-, medium-, and low-complexity classifier), on the data sets. Finally, during testing, we presented a picture to the participants and recoded the EEG signal and estimated IC. We calculated the EEG scores using the three classifiers and combined the three scores and the IC for a final score using a set of weights. We determined the category according to the final score.

#### 1) EEG module

EEG data were acquired by a g.USBamp system (G.Tec company) using 16 electrodes distributed in accordance with the international 10–20 system. The EEG data were sampled at 2400 Hz using 200 Hz low-pass and 50 Hz notch filters. Prior to scoring the images, we pre-processed the EEG data through the following steps: downsampling to 600 Hz, band-pass filtering (0.1–60 Hz) with a 10th order butterworth filter, baseline correction, and ocular artifact reduction. Here, zero- delay filtering was implemented using the filtfilt() function in MATLAB. Afterward, the EEG data were divided into epochs. Each epoch consisted of 1000 ms of EEG data after the stimulus onset.

Analysis of the ERP using HDCA algorithm was performed as described by Parra et al [13, 14, 26]. The HDCA algorithm can be divided into two layers. First, the HDCA algorithm was employed to obtain the average data and divide the original EEG data by time window size. The weight of each channel was then calculated in each time window to maximize the differences between the target and nontarget classes. In our study, the time window size cannot be determined in advance. Thus, we chose 25 ms as the time window size after numerous experimental repetitions. The weight of each channel in each time window was calculated by Fisher linear discriminant (FLD). In each time window, the EEG signal was reduced to one dimension, such as in Eq (1), as follows:

$$y_{k}=(\frac{1}{N})\sum_{n}\sum_{i}w_{ki}x_{i[(k-1)N+n]}$$ (1)

where *x*_*i*[(*k*−1)*N*+*n*]_ represents the *k*th separate time-window value from the single-trial data. The variable corresponds to the EEG activity at the data sample point *n* measured by electrode *i*. *w* is a set of spatial weights. Weight vector *w*_*ki*_ is found for the *k*th window and *i* electrode following each image presentation (*T* is the temporal resolution of the time window, *N* is the sampling time point of the time window, *F*_*S*_ is the sampling rate, *K* is the number of time window, and *n* = 1,2,⋯,*N*, *N* = *T*/*F*_*S*_, 0 ≤ *k* ≤ *K*).

$$y_{IS}=\sum_{k}v_{k}y_{k}$$ (2)

The results for the separate time windows (*y*_*k*_) are then combined in a weighted *y*_*k*_ average to provide a final interest score (*y*_*IS*_) for each image. FLD analysis was employed to calculate the spatial coefficient *w*_*ki*_, and logistic regression was adopted to calculate for the temporal coefficient *v*_*k*_. We specified a threshold greater than the threshold value, that is, a target.

In this paper, the time windows of HDCA are adjustable parameters. In order to verify the effectiveness of this TRICP, we set the time windows to be 100ms, 50ms, 33ms and 25ms respectively, and we call it Classifier I, II, III, IV, respectively.

#### 2) CV module

The CV module ranks all images through the IC. We used IC to describe the brain processing efficiency of image information. In this paper, we assumed that the human brain processing complex image is higher than the simple image. Thus, the subjects’ EEG signals induced by complex and simple target images vary. The complex and simple images are nonobjective. We aimed to use the knowledge in the CV field to accurately quantify the IC. The convolution neural network (CNN) is the most effective image classifier, with its importance partly caused by its mechanism which mimics the human brain processing of an image. The CNN is a deep neural network. At the CNN bottom layer, the image features are represented by texture, edge, structure, and other characteristics. High-layer features of CNN are often a combination of underlying features, representing more abstract semantic features. We considered that if an image containing simple semantics in the high-level net mapping, the feature weight should be focused on individual features, and irrelevant feature weights are small. Additionally, a semantic complex image (containing more semantics) will have more feature weights that are larger in CNN high-layer mapping.

Thus, the complexity of the semantic level can be described through the high-layer feature weights of the CNN. These weights of a simple semantic image are more concentrated, whereas those of a complex semantic image are more dispersed. Similarly, the complexity of an image structure can be described through the underlying feature weight distribution of a CNN model.

Therefore, we extracted the feature vector of the image in a layer of the CNN and converted this vector into IC using the following formula:

$$IC=\frac{1}{fnum}\log(\sum_{i=1}^{fnum}{\left(f_{i}\right)}^{k})$$ (3)

where *IC* is the image complexity, and *f* is the feature weight vector of the image in some layer of CNN, *fnum* is the number of characteristic features, and *k* is a parameter used to distinguish differences between high and low ICs. It is worth noting that, when *k* is greater than 1, the image complexity ranking is the same. In this paper, *k* = 2. Early studies have shown that the P300 latency and amplitude caused by different semantics images will vary. Therefore, we believe that the P300 will vary because of the different ICs. Relative to the traditional machine vision for the definition of image complexity, the Eq 3 is special and it is a meaningful innovation. In this paper, we adopted the AlexNet network proposed in [27]. The AlexNet won achieved a winning top-5 test error rate of 15.3%, compared with 26.2% achieved by the second-best entry in the ILSVRC-2012 competition. We believe that AlexNet imitates the characteristics of the human visual system, and AlexNet can be used in our study reference to some extent. We used the model trained in the caffe framework [28]. The AlexNet network consists of eight layers, and we used the fifth layer feature to calculate IC in the following analysis. We believe that features of the middle layer is reasonable, it can better combine the semantic and structural information.

**Fig 3:**
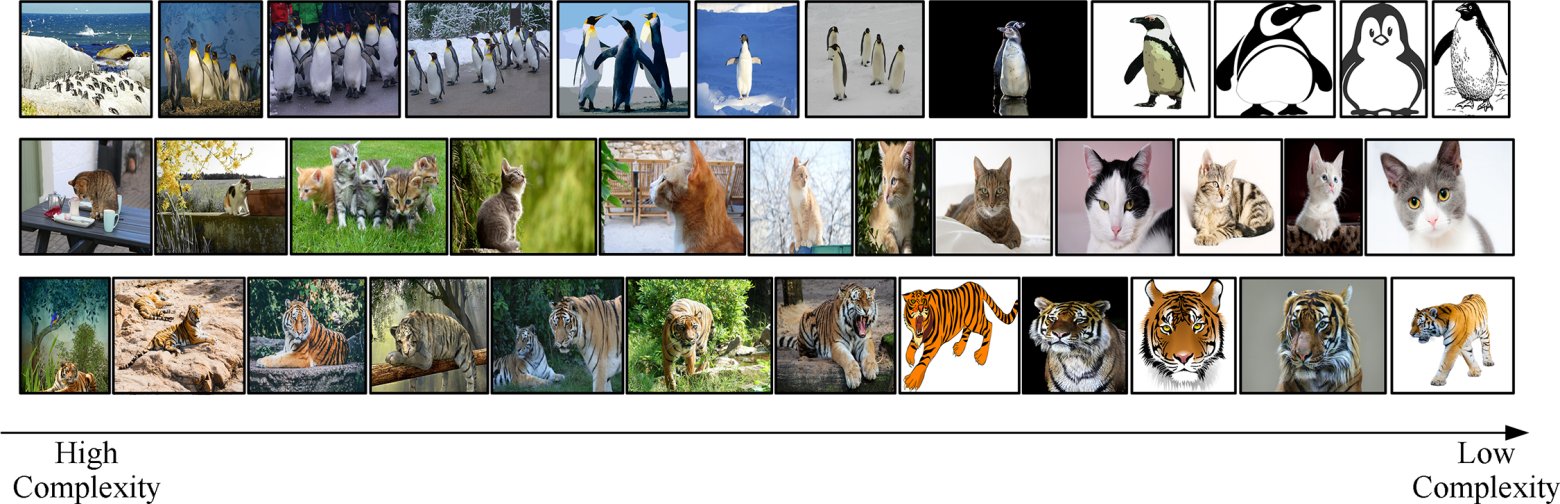
Three group images are sorted from high to low IC (The image is similar but not identical to the original image, and is therefore for illustrative purposes only).

The Fig 3 shows the results of a group of images sorted by IC.

#### 3) Mixed module

An important innovation in this study is the introduction of image information (i.e., IC) into the calculation of the EEG final score. The IC value does not contain any subjective intent (interested or not interested) when the participants viewed the picture, therefore IC cannot be directly introduced into the interest score. We used a specific fusion method as follows: the training data set was divided into three parts according to the IC, and each part train a classifier alone. During testing, the parts of interest scores were fused according to the weight into the final interest score.

The training set was divided into three parts, namely, the EEG signals from high-, medium-, and low-complexity images. Then, the corresponding training classifiers were applied independently. During testing, we calculated three interest scores of the test image using the three classifiers corresponding to the IC. The final score was combined using the following rules: the classifier weight of the test image [high IC (HIC), medium IC (MIC), or low IC (LIC)] was given a weight of *α*, and other classifier weights were assigned to *β*. This process can be expressed by Eq (4). Different classifier weights can also be assigned through a more complex process according to the IC, such as using a linear classifier (SVM, Fisher, or logistic regression) on the validation set to compute for the different classifier weights for the final score. For convenience of expression, we used a simple method.

$$score(T)=\left\{ \begin{array}{l} {\left[\alpha ,\beta ,\beta \right]}^{T}[y_{IS\_hclass}(T),y_{IS\_mclass}(T),y_{IS\_lclass}(T)],\;IC(T)\geq IC_{high\_th}\\ {\left[\beta ,\alpha ,\beta \right]}^{T}[y_{IS\_hclass}(T),y_{IS\_mclass}(T),y_{IS\_lclass}(T)],\;IC_{high\_th}>IC(T)\geq IC_{mid\_th}\\ {\left[\beta ,\beta ,\alpha \right]}^{T}[y_{IS\_hclass}(T),y_{IS\_mclass}(T),y_{IS\_lclass}(T)],\;IC(T)<IC_{mid\_th} \end{array}\right.$$ (4)

where *T* is an image, *IC*(*T*) is the IC of *T* computed by Eq (3). The *IC*_*high_th*_ and *IC*_*mid_th*_ are the IC threshold values. High IC (HIC) is obtained when the *IC*(*T*) is greater than *IC*_*high_th*_, whereas low IC (LIC) is characterized by *IC*(*T*) value less than *IC*_*mid_th*_. Values in between HIC and LIC define MIC. *y*_*IS_hclass*_(*T*), *y*_*IS_mclass*_(*T*), and *y*_*IS_lclass*_(*T*) are the interest scores computed using the high-, medium-, and low-complexity classifiers, respectively, according to the single-trial EEG induced by picture *T*. *score*(*T*) is the calculated final score combining the information from the EEG and the image. Here, *α* = 0.5, *β* = 0.25.

### Evaluation of the algorithm performance

A five-fold cross validation was conducted to determine the accuracy of all classification algorithms applied to the EEG data. Data from each subject were divided into five equal-sized trial blocks. Classifiers were trained on four of the five blocks and then tested on the remaining block. This process was repeated five times, such that each of the five trial blocks was used once as an independent testing set. Each training block used to train a classifier was divided into two parts. Performance was evaluated based on the area under the receiver operating characteristic (ROC) curve (AUC) [29].

## Results

### A. Event-related responses (targets vs. nontargets)

**Fig 4:**
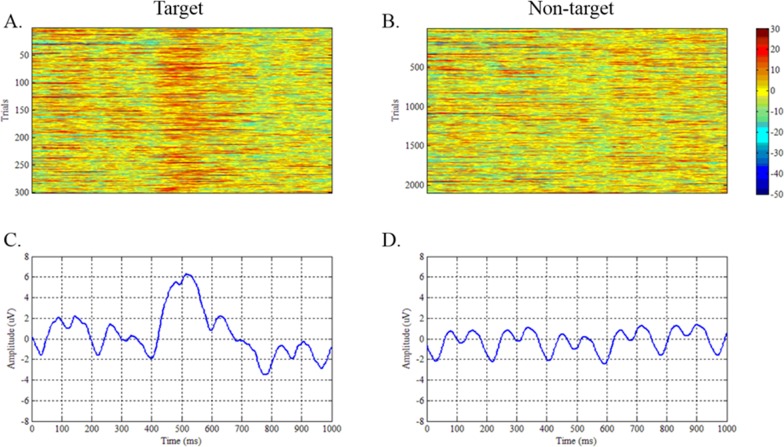
Target and non-target class waveforms. (A) All single-trial event-related potentials (ERPs) to target images at electrode Pz. (B) All single-trial ERPs to nontarget images at electrode Pz. (C) Grand averages across all trials of the target EEG signals at electrode Pz. (D) Grand averages across all trials of the nontarget EEG signals at electrode Pz.

We analyzed event-related responses to study the mean ERP, which was averaged over repeated trials under the same stimulus. Fig 4 depicts an ERP elicited by the target and nontarget ERPs at electrode Pz collapsed over blocks for a single sample subject. Fig 4 is consistent with literatures [19, 22]. Note that on an average, despite the rapid sequence of events and the overlapping responses, the main divergence between the target and nontarget ERPs occurs between 400–600 ms presentation. These results are consistent with the literature [5]. The same results can be observed with single-trial responses, as shown in Fig 4(A).

In particular, each image differed in RSVP sequence and was presented for 200 ms. Hence, the participants focused at a 5 Hz stimulus source. The EEG signals generated a mixed 5 and 10 Hz harmonic (SSVEP).

### B. Effect of IC on ERP

**Fig 5:**
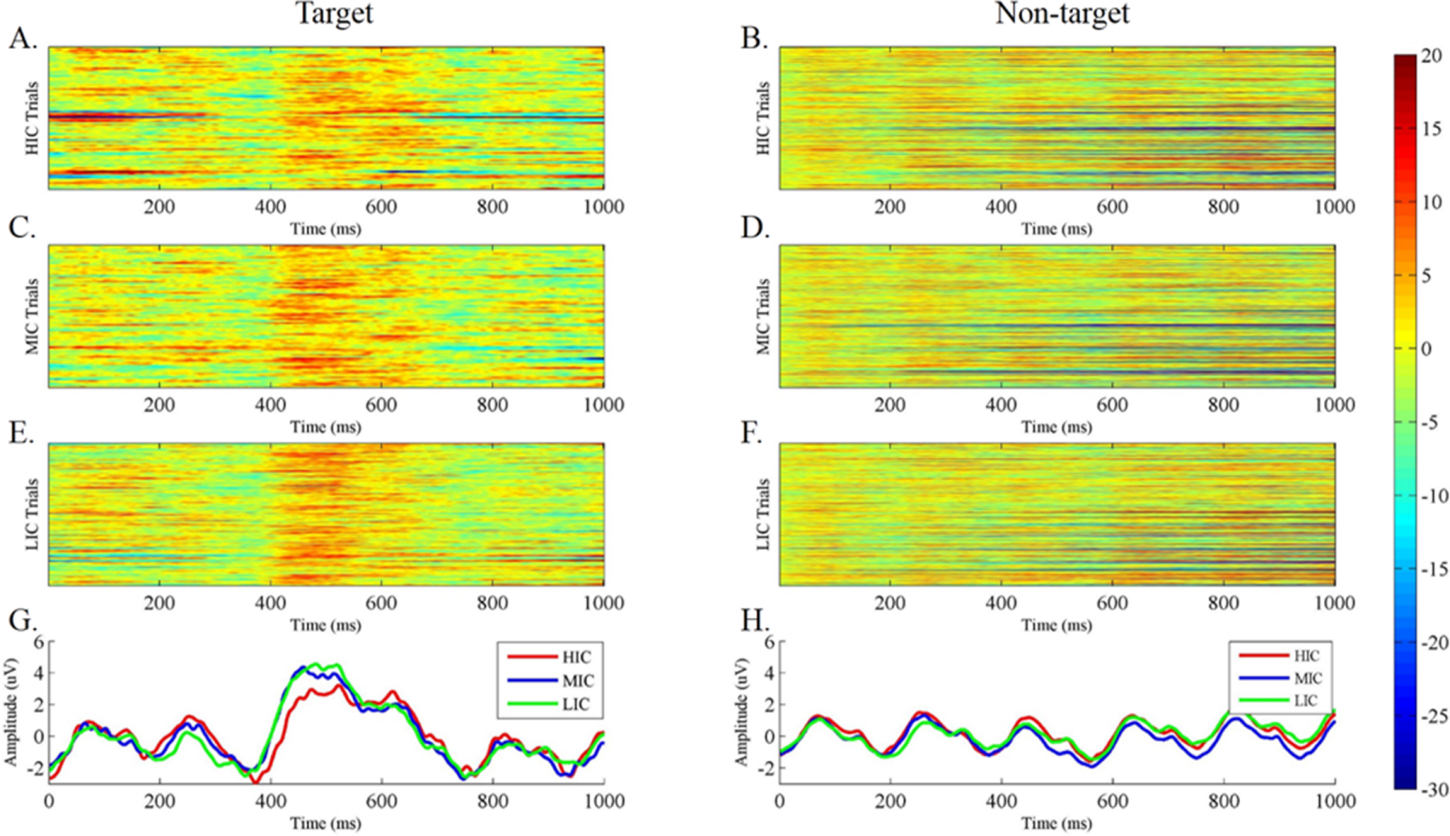
Effect of IC on ERP signal. ERPs stimulated by the (A) high-complexity target image, (B) high-complexity nontarget image, (C) medium-complexity target image, (D) medium-complexity nontarget image, (E) low-complexity target image, and (F) low-complexity nontarget image. (G) Trial averaged target ERP wave forms calculated from (A), (C) and (E), where the red, blue, and green lines indicate the ERP components inspired by high-, medium- and low-complexity image. Similarly, images in (H) are indicative of nontarget image.

To study the effect of different ICs, we averaged all subject ERP waveforms of the same image (Fig 5). We sorted all images according to IC and defined the first one-third of images as HIC, while the middle one-third is defined as MIC, and the last one-third is defined as LIC. Fig 5(A) and 5(B) show the target and nontarget ERP data caused by HIC images from electrode Pz, whereas Fig 5(C) and 5(D) show those caused by MIC images. Additionally, Fig 5(E) and 5(F) show those data caused by LIC images. The red, blue, and green lines in Fig 5(G) are the averaged ERP waveforms of the trials shown in Fig 5(A), 5(C) and 5(E), respectively. Similarly, the red, blue, and green lines in Fig 5(H) are the averaged ERP waveforms of the trials shown in Fig 5(B), 5(D) and 5(F), respectively.

Earlier studies have shown that information, such as the meaning of stimulus image, had an effect on P300 composition. Here, we illustrated the relationship through IC. Fig 5(G) shows that the amplitude of P300 excited by the HIC images was lower than that of the P300 excited by the MIC and LIC images, and the latency also varied. Fig 5(H) shows that the grand averaged ERPs of nontarget images did not significantly differ under the different IC conditions.

Table 2 shows the difference in the peak amplitudes and peak latencies of 19 participants under various IC conditions. The peak amplitude and latency were calculated using the maximum value of the averaged ERP of the different complexities. Table 2 shows that the amplitude and peak latency of HIC significantly differed from MIC and LIC (HIC, 4.76±1.09 μV; MIC, 5.44±0.9 μV; LIC, 5.49±1.22 μV; Wilcoxon Sign Rank Test, p<0.05). The amplitude of MIC did not significantly differ from that of LIC (p = 0.88). Table 2 shows that the peak latency of HIC significantly differed from those of MIC and LIC (HIC, 564.98±52.98 ms; MIC, 530.19±58.66 ms; LIC, 525.33±50.27 ms; Wilcoxon Sign Rank Test, p<0.05). The peak latency of MIC was not significantly differ from that of LIC (p = 0.52). On average, the P300 component induced by the HIC target image was 0.73 μV lower and the peak latency was delayed by 39.65 ms compared with the LIC image.

**Table 2:** The amplitude and latency changes at different complexities.

Subjects	Amplitude (μV)			Peak Latency (ms)		
	HIC	MIC	LIC	HIC	MIC	LIC
1	6.67	5.67	7.65	520.00	486.67	461.67
2	5.34	6.42	5.37	653.33	571.67	578.33
3	3.54	4.54	3.72	528.33	468.33	475.67
4	3.45	4.57	3.20	625.00	643.33	638.33
5	4.07	6.05	7.16	518.33	498.33	495.00
6	5.78	5.47	6.73	590.00	455.00	523.33
7	3.91	6.34	5.14	506.33	466.67	478.33
8	3.94	4.75	4.67	520.00	456.67	497.00
9	5.52	4.85	5.60	451.67	458.33	451.67
10	5.49	6.79	5.58	533.33	515.00	523.33
11	6.08	4.57	5.76	616.67	603.33	520.00
12	3.85	3.84	4.09	620.00	586.67	593.33
13	2.69	4.75	4.18	510.00	493.33	473.33
14	5.32	6.33	6.38	586.67	553.33	540.00
15	4.13	4.61	5.17	610.00	591.67	560.00
16	5.89	6.85	6.94	595.00	530.00	500.00
17	5.27	5.86	5.97	595.00	566.67	575.00
18	4.75	5.58	5.46	590.00	598.33	571.67
19	4.74	5.40	5.47	560.33	530.10	505.20
Mean	4.76	5.44	5.49	564.98	530.19	525.33
SD	1.09	0.90	1.22	52.98	58.66	50.27

Fali Li et al. research a relationships between the resting-state network and the P300, through a sample oddball cognitive task[30]. Fali Li et al. study indicated that P3 amplitude was significantly correlated with resting-state network topology, and no significant relationships were found for the corresponding P3 latency. However, the P300 component induced by the complex cognitive task is no more clearly conclusion. We calculated IC according to Eq (3) and the fifth layer features of the AlexNet network. We infer that the features of fifth layer showed better balance between the semantic and structural complexities. The results show that the P300 properties are different induced by different complexity images.

### C. Single-trial detection

**Fig 6:**
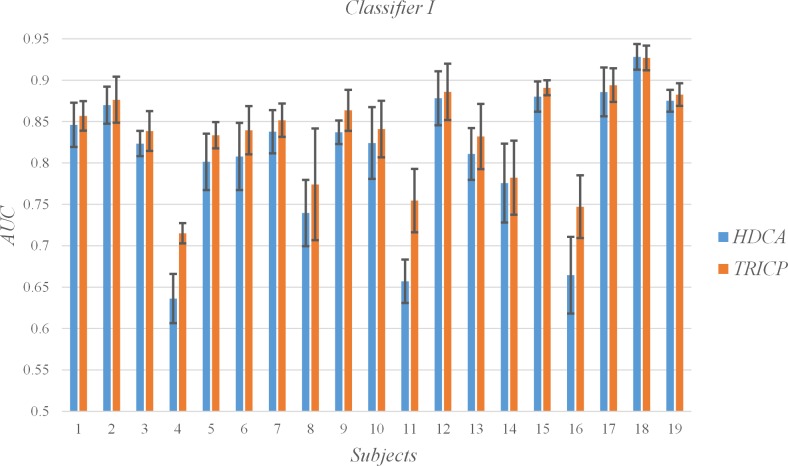
Values of the areas under the receiver operating characteristic curve (AUC) of all subjects under the two algorithms, and the time windows is 100 ms.

**Fig 7:**
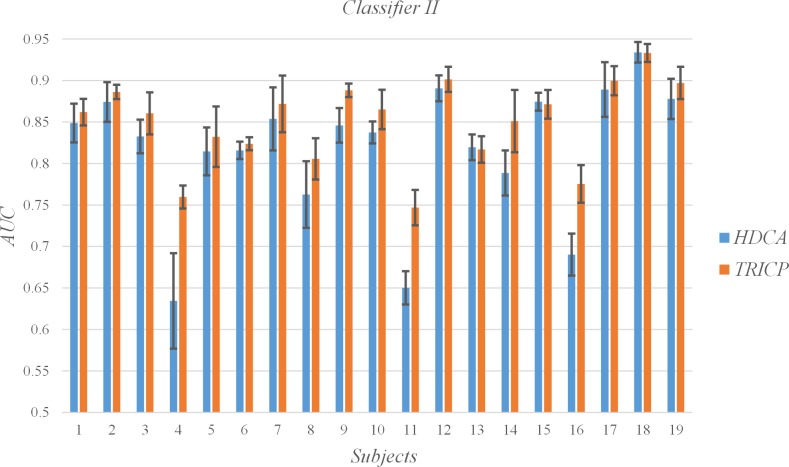
Values of the areas under the receiver operating characteristic curve (AUC) of all subjects under the two algorithms, and the time windows is 50 ms.

**Fig 8:**
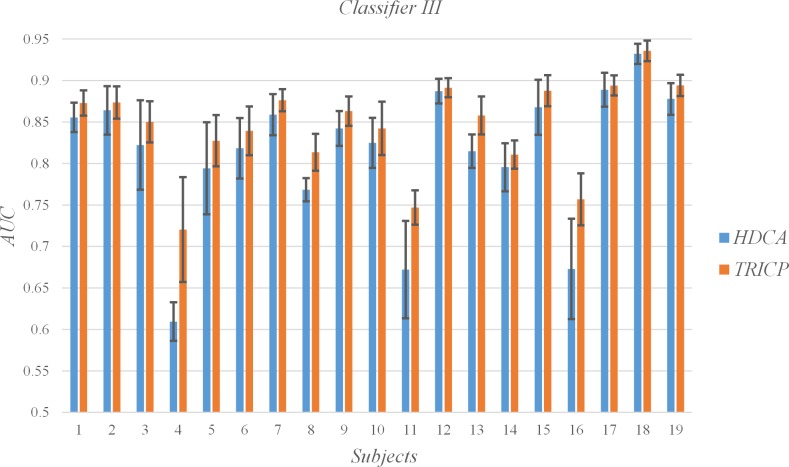
Values of the areas under the receiver operating characteristic curve (AUC) of all subjects under the two algorithms, and the time windows is 33 ms.

**Fig 9:**
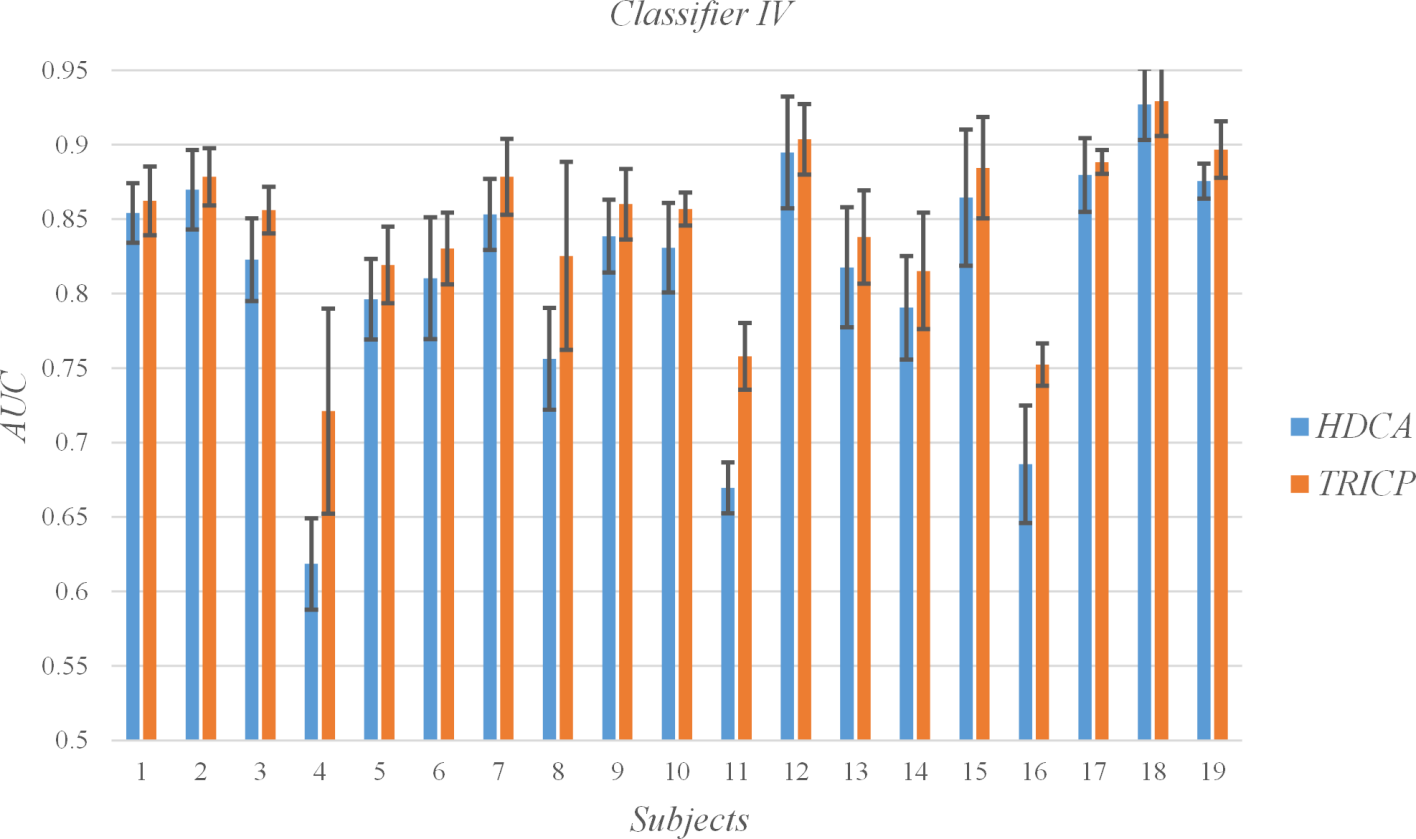
Values of the areas under the receiver operating characteristic curve (AUC) of all subjects under the two algorithms, and the time windows is 25 ms.

The AUC for each subject and the mean and SD for all subjects per algorithm group are shown in Table 3, Figs 6, 7, 8 and 9. In the experiment, each participant focused on different targets in diverse blocks (Table 1). The variation in specific meaning and complexity of the different target images led to changes in the latency and amplitude of the P300 component. The detection algorithms affected the precision of the single-trial P300, which was also demonstrated by Alpert et al.[17]. An interesting phenomenon is that TRICP achieves significantly better results than the HDCA algorithm in subjects with low AUC (e.g., Subjects 4, 11, 16). In subjects with higher AUC (e.g., Subjects 12, 18, 19), TRICP and HDCA algorithm results are similar. In subjects with moderate AUC, the TRICP better than the results of HDCA algorithm. This may indicate that, in less accurate subjects, the image is too complex may be an important reason.

**Table 3:** All subjects AUC under different classifiers.

	*Subjects*	1	2	3	4	5	6	7	8	9	10
*Classifier I*	*HDCA*	0.8461	0.8699	0.8235	0.6363	0.8014	0.8078	0.8378	0.7396	0.8372	0.8241
	*TRICP*	0.8568	0.8764	0.8386	0.7151	0.8335	0.8395	0.8516	0.7742	0.8636	0.8409
*Classifier II*	*HDCA*	0.8488	0.8743	0.8326	0.6344	0.8146	0.8159	0.8539	0.7627	0.8460	0.8375
	*TRICP*	0.8619	0.8864	0.8605	0.7597	0.8323	0.8239	0.8719	0.8057	0.8883	0.8651
*Classifier III*	*HDCA*	0.8556	0.8642	0.8223	0.6094	0.7943	0.8184	0.8590	0.7684	0.8423	0.8248
	*TRICP*	0.8729	0.8737	0.8501	0.7204	0.8275	0.8394	0.8762	0.8135	0.8632	0.8423
*Classifier IV*	*HDCA*	0.8541	0.8697	0.8227	0.6185	0.7962	0.8103	0.8532	0.7562	0.8386	0.8308
	*TRICP*	0.8623	0.8785	0.8561	0.7211	0.8192	0.8303	0.8785	0.8253	0.8600	0.8568
	*Subjects*	11	12	13	14	15	16	17	18	19	
*Classifier I*	*HDCA*	0.6572	0.8782	0.8109	0.7758	0.8802	0.6645	0.8858	0.9282	0.8751	
	*TRICP*	0.7545	0.8858	0.8320	0.7822	0.8909	0.7472	0.8940	0.9268	0.8826	
*Classifier II*	*HDCA*	0.6502	0.8906	0.8196	0.7887	0.8746	0.6903	0.8892	0.9340	0.8779	
	*TRICP*	0.7470	0.9016	0.8169	0.8512	0.8714	0.7753	0.8998	0.9333	0.8972	
*Classifier III*	*HDCA*	0.6722	0.8872	0.8148	0.7955	0.8678	0.6729	0.8890	0.9323	0.8778	
	*TRICP*	0.7470	0.8913	0.8580	0.8107	0.8877	0.7568	0.8941	0.9358	0.8942	
*Classifier IV*	*HDCA*	0.6695	0.8949	0.8177	0.7905	0.8644	0.6854	0.8796	0.9271	0.8755	
	*TRICP*	0.7578	0.9036	0.8379	0.8152	0.8846	0.7522	0.8884	0.9291	0.8968	

To solve this problem, the thesis proposed the introduction of image information. The parameter which can predigest the deformation of ERP in accordance with the IC and then target training classifier was determined. Our proposed TRICP fusion method introduces the IC of an image on the basis of an existing algorithm and improves the accuracy of single-trial ERP detection.

## Discussion

Current studies have shown that some deep neural networks process images are similar to the human brain. Agrawal et al. use the CNN based on the ImageNet image library to extract the features of the natural image, and use the middle layer as the image feature to train the fMRI visual coding model[31]. The results show that the visual coding model of CNN has achieved better prediction effect in the low-level visual area and the high-level visual area. Van Gerven et al. used a trained DNN to build a coding model to analyze the similarity between DNN and brain function brain area[32]. The experimental results show that the stimulus features exhibit hierarchical distribution on the deep neural network. Furtherly, Radoslaw et al. use a magnetoencephalography (MEG) and fMRI to observe the brain activity and compared with the DNN[33]. The results show that there is a corresponding relationship between the mapping of DNN low layer and high layer and the order of human brain vision signal processing, this together demonstrates the hierarchical structure similarity between DNN and human brain vision in spatial and temporal.

In this study, we extracted the features of an image through the different layers of the AlexNet network and converted these features to IC through Eq (3). The underlying features of the CNN network are more focused on the structural characteristics of the image. Thus, the construction can be considered as structural complexity. The features of high-level extraction of CNN network are more emphasized on the semantics of an image, such that the complexity can be regarded as semantic complexity. We believe that the characteristics of the middle layer is reasonable, it can better combine the semantic and structural information. Attention to complex images will require greater cognitive burden, and early studies have shown that the meaning of the stimulus image will affect the amplitude and peak latency of the P300 component. We carefully analyzed the P300 components and brain activity response induced by different complexity range images. We found that the brain topographic maps were different.

**Fig 10:**
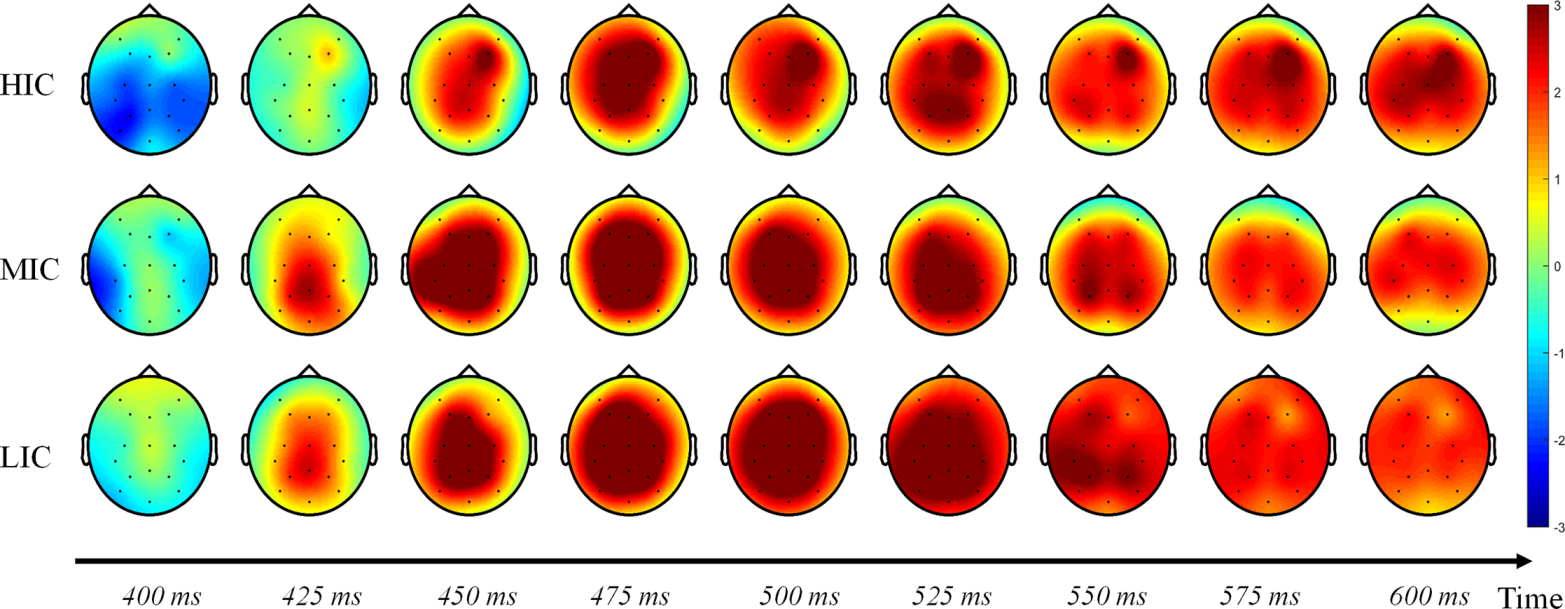
The brain activity varied between 400 ms to 600 ms in different image complexity (HIC, MIC, and LIC).

Fig 10 shows that the brain topographic maps varied between 400 ms to 600 ms in HIC, MIC, and LIC. We found a significant difference between HIC to MIC and LIC that the HIC peaked later than MIC and LIC (the HIC peaked at 475 ms, while the MIC and LIC peaked at 450 ms). This may mean that subjects need more time to identify the specific meaning of the image. In addition, between 525 ms and 600 ms, the brain activity of the LIC and MIC gradual decrease. However, in the HIC, the right frontal lobe has been active. This part is often associated with memory, semantics, images and other non-verbal ability. This result is interesting and reasonable. The subjects needed more time to analyze the specific meaning of complex images, in which case the right frontal lobe was active for a longer period of time. This result is interesting and it fits our expectations. However, due to the spatial distinguishability lack of EEG data, we cannot accurately determine the brain area of processes complex information. So combining fMRI or MEG with EEG data may be able to achieve better results, which will be the next step in the study.
